# Mild hypothermia delays the development of stone heart from untreated sustained ventricular fibrillation - a cardiovascular magnetic resonance study

**DOI:** 10.1186/1532-429X-13-17

**Published:** 2011-03-06

**Authors:** Vincent L Sorrell, Vijayasree Paleru, Maria I Altbach, Ronald W Hilwig, Karl B Kern, Mohamed Gaballa, Gordon A Ewy, Robert A Berg

**Affiliations:** 1 Department of Medicine, Sarver Heart Center, University of Arizona College of Medicine, Tucson, Arizona, USA; 2 Department of Radiology, University of Arizona College of Medicine, Tucson, Arizona, USA; 3 Department of Pediatrics, Steel Memorial Children Research Center, University of Arizona College of Medicine, Tucson, Arizona, USA

## Abstract

**Background:**

'Stone heart' resulting from ischemic contracture of the myocardium, precludes successful resuscitation from ventricular fibrillation (VF). We hypothesized that mild hypothermia might slow the progression to stone heart.

**Methods:**

Fourteen swine (27 ± 1 kg) were randomized to normothermia (group I; n = 6) or hypothermia groups (group II; n = 8). Mild hypothermia (34 ± 2°C) was induced with ice packs prior to VF induction. The LV and right ventricular (RV) cross-sectional areas were followed by cardiovascular magnetic resonance until the development of stone heart. A commercial 1.5T GE Signa NV-CV/i scanner was used. Complete anatomic coverage of the heart was acquired using a steady-state free precession (SSFP) pulse sequence gated at baseline prior to VF onset. Un-gated SSFP images were obtained serially after VF induction. The ventricular endocardium was manually traced and LV and RV volumes were calculated at each time point.

**Results:**

In group I, the LV was dilated compared to baseline at 5 minutes after VF and this remained for 20 minutes. Stone heart, arbitrarily defined as LV volume <1/3 of baseline at the onset of VF, occurred at 29 ± 3 minutes. In group II, there was less early dilation of the LV (p < 0.05) and the development of stone heart was delayed to 52 ± 4 minutes after onset of VF (P < 0.001).

**Conclusions:**

In this closed-chest swine model of prolonged untreated VF, hypothermia reduced the early LV dilatation and importantly, delayed the onset of stone heart thereby extending a known, morphologic limit of resuscitability.

## Introduction

Therapeutic hypothermia has been used since the 1950 s to mitigate neurological injury from cardiac arrest. Most of these investigations focused on moderate hypothermia (28°C-32°C), deep hypothermia (< 28°C), and profound hypothermia (< 15°C) for neuro-protection [1,2]. These deep levels of hypothermia have been used to provide safer open-heart surgery, presumably because of substantial reduction in neuronal and myocardial oxygen requirements. However, they can cause ventricular fibrillation (VF) and coagulopathy, and if prolonged, can lead to life-threatening infections [3]. In contrast, mild hypothermia (32°C-34°C) is safer and provides neuro-protection from ischemia and ischemia-reperfusion injuries [1,2]. Two randomized controlled clinical trials established that mild hypothermia improved outcome for comatose adults after resuscitation from VF cardiac arrest [4,5]. Mild induced hypothermia is now the recommended treatment for persistently comatose adults after resuscitation from out-of-hospital VF cardiac arrest [5]. It should be noted that most studies regarding the benefits of induced hypothermia for ischemia and ischemia-reperfusion insults have focused on neuro-protection [1,2,6,7]. The cardio-protective benefits of induced mild hypothermia are less well studied. Recently, it was demonstrated that mild hypothermia was able to significantly improve defibrillation success and resuscitative outcomes after 8 minutes of sustained VF [8]. However, the mechanisms of this benefit were not explained, although it was shown not to be dependent on coronary artery perfusion pressure.

*Stone Heart *is the global ischemic contracture resulting in firm myocardium and loss of LV intracavitary volume - so called *myocardial rigor mortis *[9]. An early complication of cardiopulmonary bypass, known to cardiac surgeons to represent the inability for successful resuscitation, it is now largely prevented with adequate cardioplegia [10-12]. Although stone heart has been described in animals and humans, laboratory investigations of stone heart have focused on acute aortic cross-clamp ischemic models and on *in vitro *models [9-14]. The stone heart phenomenon has not been extensively studied in closed-chested, whole animal models of sustained VF. Our laboratory was the first to study stone heart in these animal models with minimal intra-thoracic surgical interventions by using cardiovascular magnetic resonance (CMR) [15,16]. Furthermore, although induced mild hypothermia is a well-established effective therapy for improving neurological outcomes, its effects on early ventricular dilation or late stone heart development, secondary to untreated VF, are unknown. Therefore, this study was designed to test the hypothesis that in a closed chest animal model of VF cardiac arrest, pre-arrest induced mild hypothermia substantially extends the time interval from commencement of cardiac arrest until the development of stone heart.

## Methods

### Animal Preparation

Before this experimental protocol was initiated, approval was obtained by the University of Arizona Institutional Animal Care and Use Committee and has been previously reported in detail [17]. Fourteen female swine (27 ± 1 kg) were used in this study. Animals were randomized to two groups, group I (normothermia, n = 6) and group II (hypothermia, n = 8). Animals were anesthetized with a mixture of room air and titrated isoflurane (≤ 1% to 2.5%), followed by oral endotracheal intubation. Animals were mechanically ventilated with a rate- and volume-regulated ventilator (Narkomed 2A, North American Drager) and the end-tidal carbon dioxide was maintained at 40 ± 2 mmHg. After a surgical plane of anesthesia was obtained, introducer sheaths were placed in the right internal jugular vein and right carotid artery by cut-down technique. A high-fidelity, solid-state, micromanometer-tipped catheter (MPC-500, Millar Instruments) was advanced into the thoracic aorta for initial pressure monitoring.

### Experimental Protocol

Prior to CMR imaging, mild hypothermia (32-34°C) was induced by surrounding the swine head, thorax and abdomen with ice packs to achieve a rectal temperature of 32-34°C. Routine CMR scout images in normal sinus rhythm were obtained allowing the optimal alignment for accurate ventricular volume calculations. Detailed methodology has been previously reported [17]. A pacing catheter electrode was placed temporarily into the right ventricle (RV). Ventricular fibrillation was induced with 100-Hz alternating current delivered in the RV and confirmed by the ECG waveforms. Assisted ventilation was discontinued, and the pacing wire was removed.

### Measurements

CMR was obtained using a 1.5-T GE Signa NV-CV/i scanner (GE Medical Systems). A 4-element phased-array coil was used for signal detection. Data were acquired with a steady-state free-precession pulse sequence. Pulse sequence parameters were as follows: repetition time, 3.7 ms; echo time, 1.6 ms; α = 45°; acquisition matrix size, 224 × 224; field of view, 36 × 27 cm^2^; slice thickness, 6 mm; and slice gap, 0 mm. Before onset of VF, a short-axis stack of images were acquired at multiple phases throughout the cardiac cycle with retrospective ECG gating. Complete anatomic coverage of the heart from just beyond the cardiac apex through the entire ventricular myocardium was acquired. After VF, the pulse sequence was set to acquire ungated data. In this manner, data through the same exact slices were acquired in approximately 10 seconds (acquisition time per slice, 658 ms). Data collection started immediately after the initiation of VF, every minute for the first five minutes, and every five minutes thereafter until the development of stone heart was visibly observed (or a maximum of 35 minutes in group I or 120 minutes in group II).

### Off-line CMR data analysis

CMR image data were transferred to an off-line workstation for volume calculation (GE ReportCARD, Cardiac MR analysis software). The interpreter was blinded to the assigned group. The endocardium of the LV and RV was manually traced at each time point and the LV and RV volumes were calculated from the summed total of the individual slice volumes using Simpson's method as previously described [15]. More than 10,000 individual images were analyzed. Stone heart was arbitrarily defined as a loss of 2/3 of LV volume compared to baseline volume at the onset of VF, because this endpoint was easily measurable and invariably occurred in close temporal proximity to the minimal volume attained in each animal.

### Statistical analysis

Data are described as mean ± standard error (SE). All statistical analyses were performed with Stata 8 (Stata Corp LP) and StatView 5.0 (SAS Institute, Inc) software. Comparisons of mean LV and RV volumes were obtained as continuous variables using ANOVA with repeated-measures. Baseline was considered as initial CMR measurements immediately after initiation of VF. Table 1 depicts the LV and RV volumes at 5 minute increments in both groups. For statistical analysis purposes, "time to stone heart" is defined arbitrarily in this study as the time required to reach 2/3 reduction in LV volume compared to baseline. For all statistical analyses, a p < 0.05 was considered significant.

**Table 1:** 

	Hypothermia (n = 8)		Normothermia (n = 6)	
Time (min)	LV (ml)	RV (ml)	LV (ml)	RV (ml)
**0**	40 ± 3	46 ± 3	40 ± 5	51 ± 5
**5**	45 ± 3*	62 ± 5	48 ± 3	67 ± 4
**10**	46 ± 4	62 ± 5	47 ± 3	65 ± 4
**15**	44 ± 4	62 ± 6	47 ± 2	65 ± 3
**20**	43 ± 5	60 ± 6	46 ± 2	62 ± 3
**25**	40 ± 8	58 ± 6	33 ± 7	59 ± 3
**30**	40 ± 6*	56 ± 6	12 ± 1	51 ± 2
**35**	36 ± 8*	50 ± 8	---------	---------
**40**	31 ± 5*	49 ± 7	---------	---------
**45**	19 ± 3*	41 ± 4	---------	---------
**50**	14 ± 2*	37 ± 4	---------	---------
**55**	12 ± 2	34 ± 4	---------	---------
**60**	12 ± 2	34 ± 4	---------	---------

Changes in ventricular dimensions during normal sinus rhythm and during VF in both normothermia and mild hypothermia groups. Data are mean ± SE, *p < 0.05 hypothermia vs normothermia.

## Results

There was no difference in the LV and RV volumes at baseline (Table 1; time 0). Although the final LV volume at the end of the stone heart experiment was somewhat variable, it decreased in all cases to <1/3 of baseline (e.g. all developed stone heart) and from this point onward there was very minimal additional change in ventricular volumes or myocardial thickness.

**Figure 1:**
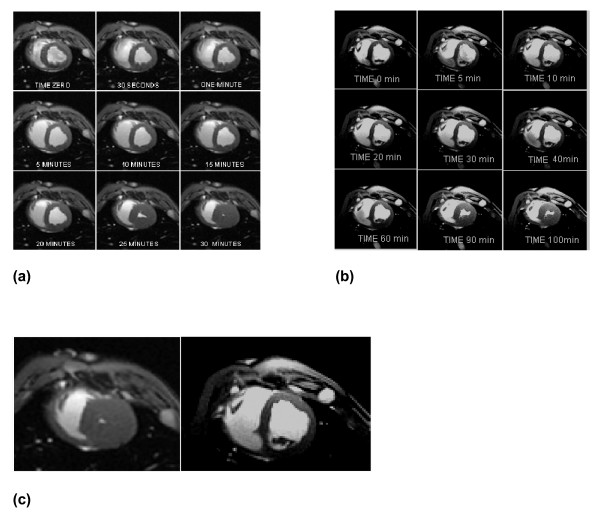
**Cardiac Magnetic Resonance images of the swine heart at the level of the mid-ventricular short axis view of the right and left ventricles**. **a**) A representative normothermia animal over 30 minutes of untreated ventricular fibrillation. Stone heart in this example developed at ~25 minutes; **b) **A representative hypothermia animal over 100 minutes of untreated VF. Stone heart in this animal developed at ~90 minutes. Time 0 refers to time ventricular fibrillation (VF) was induced, and next 8 views were at respectively labeled duration of untreated VF; **c) **MRI "zoom" comparison of the 30 minute image for the representative normothermia animal (left) and the hypothermia animal (right).

**Figure 2:**
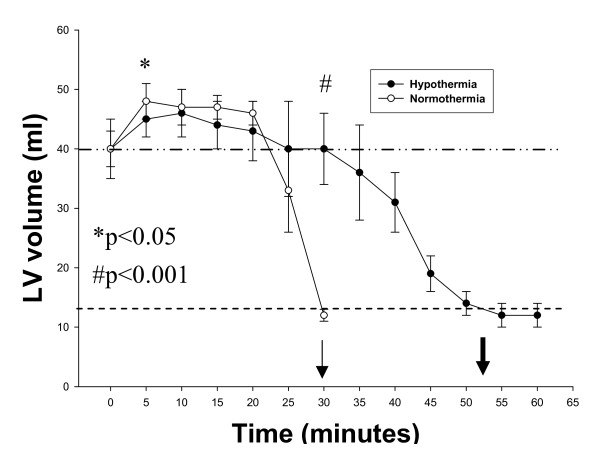
**The time course of mean left ventricular (LV) volumes (mean ± SEM) during untreated VF in both normothermia and hypothermia swine**. *Ventricular volume differs between the two groups with less dilation of hypothermia group (p < 0.05). #Ventricular volume differs between the two groups with less contracture in the hypothermia group (p < 0.001). The dashed and dotted line represents baseline LV volumes at VF onset. The even dashed line represents LV volume at stone heart. The thin arrow represents time to stone heart in normothermia group. The thick arrow represents time to stone heart in hypothermia group.

A CMR short axis view of LV and RV at the mid-ventricular slice during 30 minutes of untreated VF for a representative normothermia animal is shown in Figure 1a. In striking uniformity with our previous reported findings in untreated VF in normothermia swine, there is a substantial increase in left ventricular septal and posterior myocardial wall thickness at 25 minutes and the LV volume is essentially obliterated at 30 minutes. Figure 1b is a CMR short axis view from a representative hypothermia animal during 100 minutes of untreated VF. In contrast to Figure 1a, at 30 minutes of untreated VF, hypothermia animals had no change in LV volume compared to baseline (at the onset of VF). Time to stone heart occurred at 52 ± 4 minutes in the hypothermia animals compared to 29 ± 3 minutes in the normothermia animals (81% delay; p < 0.001, Figure 2, and Table 1). Ventricular measurements during normal sinus rhythm and during VF are included in Table 1. The mean values of LV volumes for both groups are shown in Figure 2.

During the first 5 minutes of untreated VF, mean LV volume increased by 11% from baseline values in the hypothermia group compared to 34% increase in the normothermia group (p < 0.05). Between 10 and 30 minutes of untreated VF, there were no significant changes in LV volumes in the hypothermia group versus a 75% decrease in LV volume in the normothermia group (p < 0.05).

**Figure 3:**
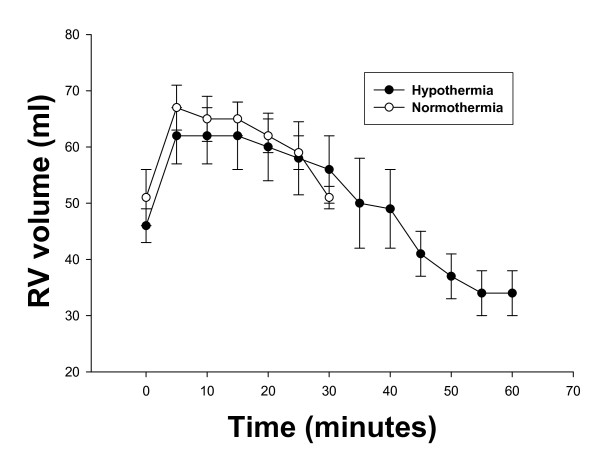
**The time course of mean right ventricular (RV) volumes (mean ± SEM) during untreated ventricular fibrillation (VF) in both normothermia and hypothermia swine**. Note that the right ventricle never approaches 2/3 of baseline value - never achieves stone heart.

The mean values of RV volumes for both groups are shown in Figure 3. In contrast to the LV volume, in both groups, mean RV volume increased by 35% from baseline within the first 5 minutes of untreated VF. No differences were observed between the two groups during these first 5 minutes of VF. It should be noted that RV volume never decreased below 30% of the baseline VF value.

## Discussion

The major findings of this CMR study are that inducing mild hypothermia (32-34°C) by external application of ice prior to the onset of sustained VF causes a characteristic and reproducible reduction in early LV dilatation and importantly, a delay in the development of stone heart compared with normothermic controls. This is the first study to demonstrate a proposed direct structural or anatomic mechanism as a marker of the metabolic state or milieu of the LV myocardium for the known beneficial consequence of mild hypothermia. The use of CMR for this research has proven valuable in obtaining highly reproducible results and allowing the investigation to occur in closed chest, whole heart swine models using commercially available scanners [17]. Given the excellent spatial resolution of CMR, the comprehensive anatomic data sets, and the test-retest accuracy of this imaging modality, extensive data can be obtained using fewer animals.

The current strategies for treatment of cardiac arrest are prompt defibrillation for the shockable rhythms of ventricular fibrillation or pulseless ventricular tachycardia (VF/VT), and prompt basic life support for the non-shockable rhythms of asystole and pulseless electrical activity. When VF/VT is more prolonged (> 4-6 minutes), pre-shock and/or post-shock chest compressions are generally necessary for successful resuscitation (the *circulatory phase *of VF) [18]. We and others have shown that pre-shock CPR in both experimental models and clinical studies can improve outcome from prolonged VF during the *circulatory *phase [19,20]. Data from our laboratory also established that immediate CPR after countershock is another successful strategy for improving outcome in this *circulatory *phase of VF [21].

When VF is even more prolonged (> 10-15 minutes), outcomes are generally poor. According to the 3-phase time-sensitive model of untreated VF proposed by Weisfeldt and Becker, this encompasses the *metabolic phase *[18]. Some experts have recommended discontinuing resuscitation efforts after as little as 10-15 minutes because of these poor outcomes [22]. Moreover, international guidelines note that prolonged resuscitative efforts are unlikely to be successful and should be discontinued after 30 minutes without return of spontaneous circulation [23].

Once spontaneous circulation returns, current recommendations are for the use of hypothermia in post-cardiac arrest coma patients. A growing trend in the use of hypothermia is now seen and the probability that recurrent VF will occur after hypothermia must be considered. Therefore, our findings are encouraging and partially help to provide a mechanistic etiology to earlier demonstrable benefits [8].

Additionally, good outcomes have been reported despite prolonged cardiac arrests in ice-water drowning events - an important clinical scenario that may parallel the findings in this report [3,24-26]. These victims likely have not only a neuroprotection, but a direct cardioprotective benefit previously not considered. Numerous reports exist that demonstrate the ability to successfully resuscitate cold water drowning victims after remarkably prolonged periods of submersion [24]. Usually these are extremely cold water cases and most commonly involve children with a larger surface to mass ratio. It is likely that these findings reflect the need to obtain relatively rapid cooling to achieve maximal benefit. These cases challenge our concepts of the alleged limits of cardiac and cerebral anoxia, which are usually accepted as 15-20 and 5-7 minutes, respectively.

To further improve survival from cardiac arrest it is important first to determine the practical limit of myocardial resuscitability and design interventions to extend this limit. While investigating the pathophysiology of the circulatory phase of VF with CMR studies, we observed the reproducible development of stone heart after 25-35 minutes of untreated VF in normothermic swine. Stone heart appears to be an irreversible phenomenon and is thereby a presumed limit of myocardial resuscitability that prevents effective CPR (i.e., the heart is refractory to efforts at perfusion secondary to collapse of the mircrocirculation from the tetanic systolic contraction associated with the ischemic myocyte contracture) [8,9].

Stone heart, or ischemic myocardial contracture, was characterized by Cooley et al as contracture of the myocardium and inability to obtain cardiac output on manual massage [27]. They recognized the fatal nature of this rare condition. Furthermore, they noted more than 30 years earlier that prevention of stone heart could be afforded with topical and general hypothermia. Takino and colleagues identified 36 patients with stone heart - described in their work as 'firm myocardium' [28]. The 'very firm' group never resumed contraction after treatment, whereas the 'less firm' (not 'soft') group showed some, albeit insufficient, contractile activity supporting that this represents a limit of resuscitability. These investigators noted that the firm myocardium was *"mainly in the left ventricle"*. This is consistent with what was found in our closed chest CMR model of stone heart where at the point of LV stone heart onset (Figure 3), the RV volume decrease appeared to plateau.

The mechanisms of hypothermia-mediated delay of stone heart development are unclear, but likely reflect some attenuation of ischemic injury. Hypothermia has been demonstrated to modify multiple steps in the ischemic cascade that in total seem to ameliorate severe ischemic myocardial injury. There is a direct relationship between canine myocardial temperature and ischemic ATP decay. Hypothermia was shown to delay the time interval of ischemia during which ATP content decreases from normal values to the presumed 'energy limit of resuscitability' and hypothermia was demonstrated to delay the reduction of pH [29]. This work supports the hypothesis that hypothermia exerts at least some of its beneficial effects by limiting ATP loss in the myocardium and/or by slowing the onset of marked acidosis.

In surgery, where stone heart was first described, cardioplegia solutions are utilized to slow the rapid decay of ATP. Importantly, when combined with hypothermia, their protective efficacy is intensified. In a rabbit model of coronary artery occlusion and reperfusion, infarct size was strikingly reduced (65%) by a 2-4 degrees C reduction in direct myocardial temperature using topical hypothermia [30]. Inducible nitric oxide synthase (iNOS) is known to contribute to both myocardial and cerebral ischemic injury and hypothermia has been shown to decrease iNOS activity[31].

These findings have direct clinical impact. Previous studies showed that when VF was induced for 8 minutes in the setting of moderate or severe hypothermia, the ability to resuscitate was improved [8]. There was an increase in defibrillation success and survival and a decrease in the risk of refibrillation. These beneficial effects of hypothermia were not due to alteration of coronary perfusion pressure. Also, there are variable effects of hypothermia on defibrillation energy requirements lowering the likelihood that this benefit is strictly electrophysiologic [32]. Therefore, the major benefit of hypothermia is more probably due to an intrinsic cellular and/or biochemical change within the myocardium, which leads to changes in the myocardial mechanical properties. Our findings that hypothermia delays the early LV dilatation may explain, at least in part, the increased defibrillation success and resuscitation outcomes previously reported at 8 minutes of untreated VF [8]. Defibrillation success and resuscitation outcomes in more prolonged untreated VF have yet to be studied with hypothermia. At 8 minutes of sustained VF, our earlier work, confirmed in the normothermic group in this study, demonstrated significant increases in the LV and RV volumes. Our hypothermia group had this structural alteration attenuated which may have contributed to the defibrillation success.

It has been suggested that VF would result in a rapid energy depletion and profound ischemia that would negate any potential beneficial effects of hypothermia. Our findings may alter that notion by demonstrating that even VF may benefit from hypothermia. It is possible that the documented cases of prolonged CPR after accidental hypothermia profited from the cardioprotective effects in addition to the presumed neurocognitive protection of hypothermia.

Current clinical practice recommends the induction of hypothermia after cardiac arrest survivors in coma. However, *intra-arrest cooling *is being investigated as a potential novel beneficial option to improve survival from VF arrest and technology may soon offer devices for safe, rapid hypothermia induction [33]. If our findings in the swine model translate to the human, then we expect that this technique will provide incremental benefit given the established ability of hypothermia to postpone the progression to the presumed final limit of resuscitability. Recent work has revealed that hypothermia during *intra-arrest cooling*, had a greater survival than normothermia, or those with delayed hypothermia [34]. The strategy of improving survival from cardiac arrest by extending the limit of resuscitability is an intriguing one. The development of stone heart is presumed to be the limit of resuscitability from cardiac arrest. Extending this limit using hypothermia, as shown in our study, may be an important clinical application with significant beneficial ramifications.

## Limitations

As with all animal studies, caution is warranted with regard to direct extrapolation to humans. The time course of these phenomena among humans in VF may be different. No pathology or blood work was available on these animals and gross or microscopic cellular changes cannot be confirmed.

Temperature was measured with a rectal thermometer and this may have varied from the core temperature. No attempt to maintain mild hypothermia during untreated VF was provided and therefore, the animals' temperature likely decreased during the CMR examination. This may have influenced the reported findings, but could not account for the noted differences between groups. The duration of pre-arrest anesthesia was not controlled between the two groups and the hypothermia group had by necessity a longer period of anesthesia than the normothermia group. This is known to impact ventricular contraction, but its influence on our findings in hypothermia cannot be determined.

In this study, hypothermia was initiated pre-arrest and this is not currently feasible in most clinical settings. However, newer and much more rapid techniques of mild hypothermia induction are in development [35,36]. Moreover, these findings may add insight into the analogous phenomenon of delayed resuscitation from accidental hypothermia during ice-water drowning.

## Conclusion

Most of the focus on the benefits of hypothermia during prolonged CPR has been on the lower metabolic demands of the brain with an oxygen-sparing value. Little mention of the cardiac benefits of hypothermia has been raised. Given the clearly demonstrated delay in initial LV dilation and the markedly prolonged onset to stone heart in our whole heart animal model, it seems that a cardiac benefit is genuine and may be equally important to the favorable neuro-metabolic role.

Our data is the first to demonstrate that in a closed-chest whole animal model of untreated prolonged VF cardiac arrest, pre-arrest induced mild hypothermia substantially extends the time interval from commencement of cardiac arrest until the development of stone heart. The pathophysiologic mechanism of these reported findings remains speculative and warrants additional investigation.

## Competing interests

The authors declare that they have no competing interests.

## Authors' contributions

VS performed the CMR exam, oversaw the imaging analysis, and composed the final accepted manuscript. VP carried out the preliminary CMR analysis and performed the initial statistical assessments. MA created the CMR sequences and optimized the image quality. RH oversaw the animal care and instrumentation throughout the experiment. KK and GE coordinated the entire investigative team, assisted with study design, and greatly participated in manuscript drafts. MG wrote the initial draft and performed the initial published data review. RB conceived of the study, participated in its design and coordination, and completed the final statistical analysis.
